# Integration of AI-Generated Images in Clinical Otolaryngology

**DOI:** 10.7759/cureus.68313

**Published:** 2024-08-31

**Authors:** Ramin Javan, Jamie Cole, Sabrina Hsiao, Brennan Cronquist, Ashkan Monfared

**Affiliations:** 1 Department of Radiology, George Washington University School of Medicine and Health Sciences, Washington, USA; 2 Department of Otolaryngology – Head and Neck Surgery, George Washington University School of Medicine and Health Sciences, Washington, USA

**Keywords:** technology-enhanced education, ai-generated images, artificial intelligence in medicine, ai-assisted clinical diagnosis, image generation, text-to-image, clinical otolaryngology, generative artificial intelligence, dall-e 3, midjourney

## Abstract

Recent advances in generative artificial intelligence (AI) have enabled remarkable capabilities in generating images, audio, and videos from textual descriptions. Tools like *Midjourney* and *DALL-E 3* can produce striking visualizations from simple prompts, while services like *Kaiber.ai* and *RunwayML Gen-2* can generate short video clips. These technologies offer intriguing possibilities for clinical and educational applications in otolaryngology. Visualizing symptoms like vertigo or tinnitus could bolster patient-provider understanding, especially for those with communication challenges. One can envision patients selecting images to complement chief complaints, with AI-generated differential diagnoses. However, inaccuracies and biases necessitate caution. Images must serve to enrich, not replace, clinical judgment. While not a substitute for healthcare professionals, text-to-image and text-to-video generation could become valuable complementary diagnostic tools. Harnessed judiciously, generative AI offers new ways to enhance clinical dialogues. However, education on proper, equitable usage is paramount as these rapidly evolving technologies make their way into medicine.

## Editorial

While the use of artificial intelligence (AI) across the medical field has made significant progress, AI-powered image and video generators have yet to be explored as useful tools. Midjourney and DALL-E 3 are two of several newly developed AI programs. Familiarity with visual arts can enhance observational skills, allowing clinicians to detect subtle changes in patient presentations, and recent studies have explored the use of AI-generated art in medicine [1]. Moreover, art encourages empathy and cultural sensitivity, enabling better patient-clinician communication [2]. Furthermore, medical illustrations and visual aids simplify complex concepts, facilitating clearer understanding and informed decision-making [3]. Thus, art can serve as a bridge between the scientific and humanistic aspects of patient care.

For this submission, Midjourney v5.2 was accessed through the Discord platform, where after adding the Midjourney server, images could be generated on any of the #general server channels by using the “/imagine” command in the text prompts. There are parameters that can be added to the end of a prompt following two dashes "--" in order to customize the generated images (Table 1). It is possible to provide an initial image as a starting point to be combined with the text prompt. Midjourney 6 has since become available with a more user friendly inerface directly on its website. DALL-E 3, released in early October 2023, is accessible through ChatGPT. It has a simple user interface and images can be interactively modified by simply conversing with the chatbot.

**Table 1:** Useful parameters in Midjourney v5.2

Parameter	Command	Function
Aspect Ratio	--ar	Specify aspect ratio in the format of a:b width-to-height ratio (where "a" is the width and "b" is the height). The default value is 1:1.
Image Weight	--iw <0-2>	Sets image prompt weight relative to text weight. The default value is 1.
Exclude	--no	Negative prompting in order to exclude an entity.
Realistic Style	--style raw	Photorealistic style of image generation.
Seed	--seed	Using the same seed number from any image generation will produce similar ending images.
Chaos	--chaos <0-100>	Changes how varied the results will be, with higher values producing more unusual and unexpected generations.
Repeat	--repeat <1-40>	Create multiple jobs from a single prompt.

**Figure 1:**
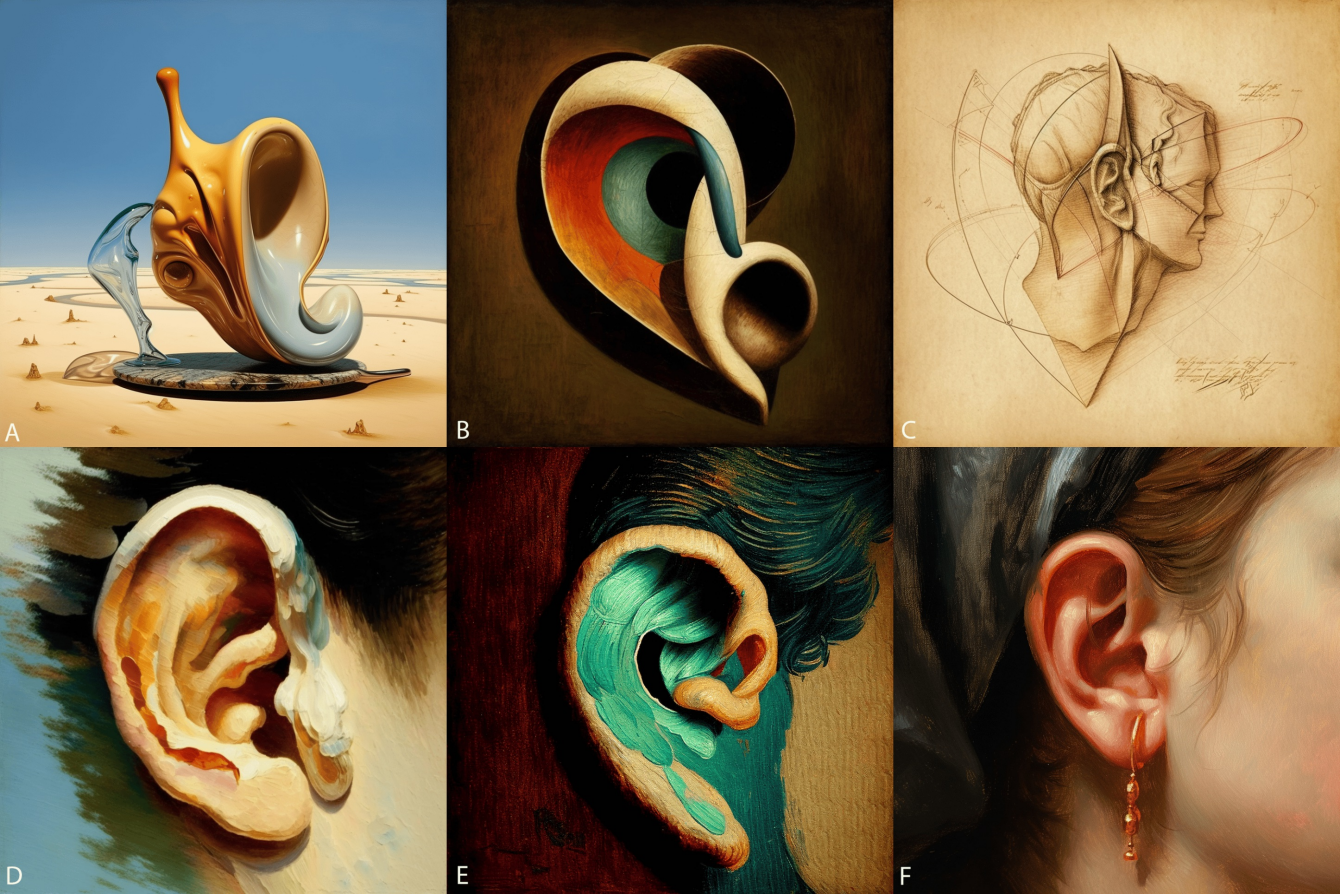
Outer ear art (images generated by Midjourney) From top left to bottom right: the ossicles in the style of A) Salvador Dali, B) Picasso, C) Leonardo Da Vinci, D) Claude Monet, E) Van Gogh’s Starry Night, and F) Rembrandt.

**Figure 2:**
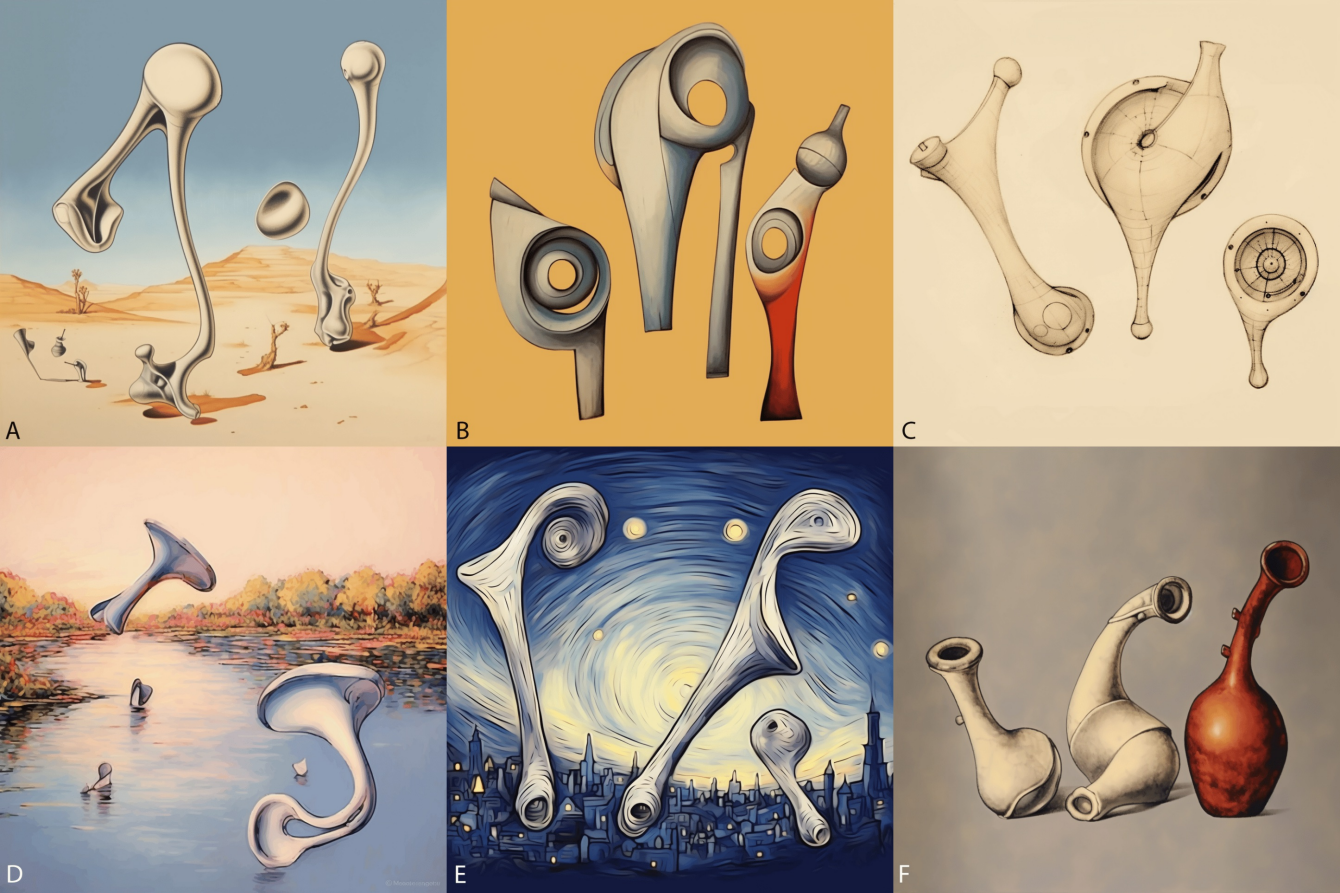
Middle ear art (images generated by Midjourney) From top left to bottom right: the ossicles in the style of A) Salvador Dali, B) Picasso, C) Leonardo Da Vinci, D) Claude Monet, E) Van Gogh’s Starry Night, and F) Rembrandt. An initial sketch of the middle ear ossicles was provided to Midjourney for proper results.

**Figure 3:**
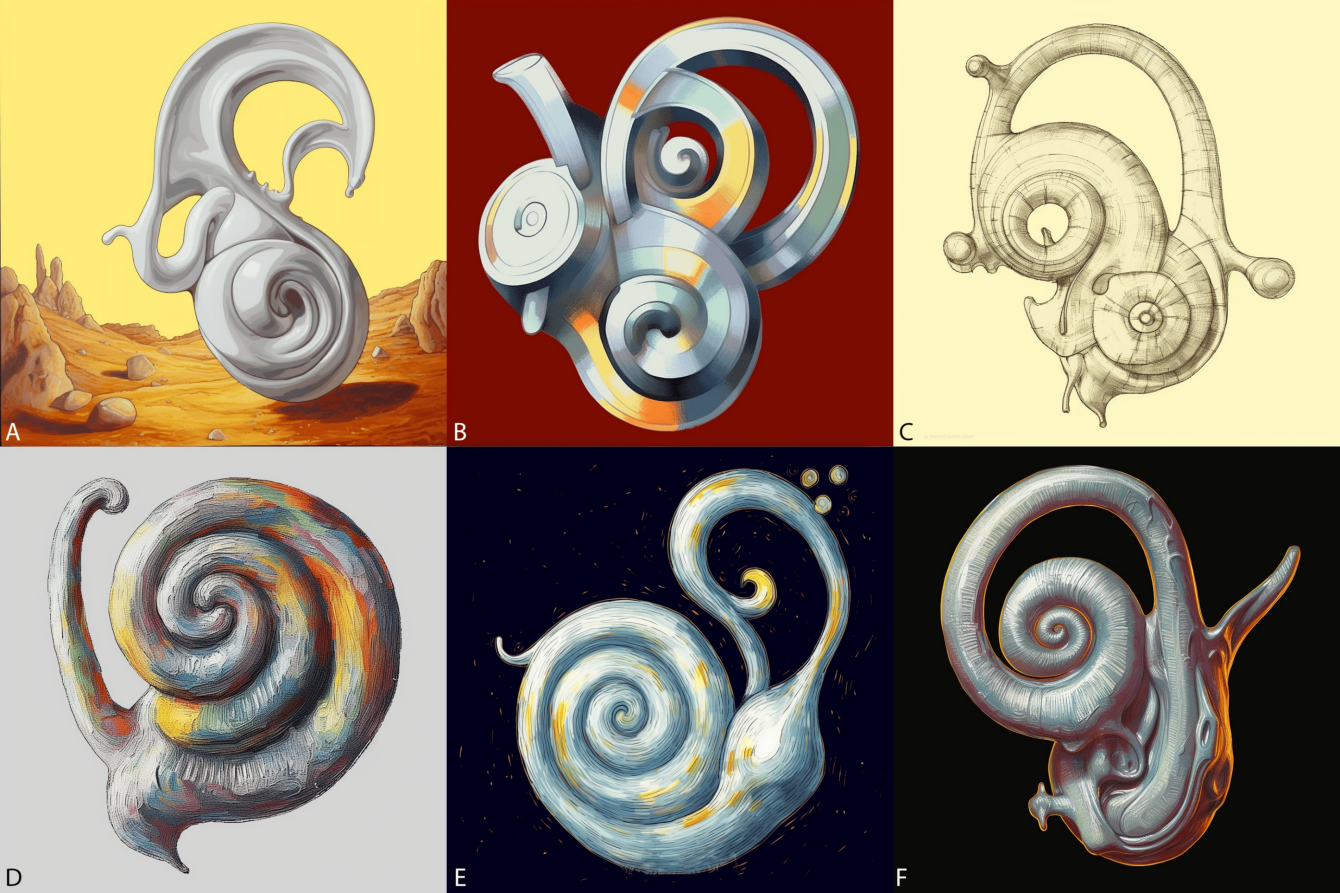
Inner ear art (images generated by Midjourney) From top left to bottom right: the inner ear in the style of A) Salvador Dali, B) Picasso, C) Leonardo Da Vinci, D) Claude Monet, E) Van Gogh’s Starry Night, and F) Rembrandt. An initial sketch of the inner ear structures including the cochlea and semicircular canals were provided to Midjourney.

**Figure 4:**
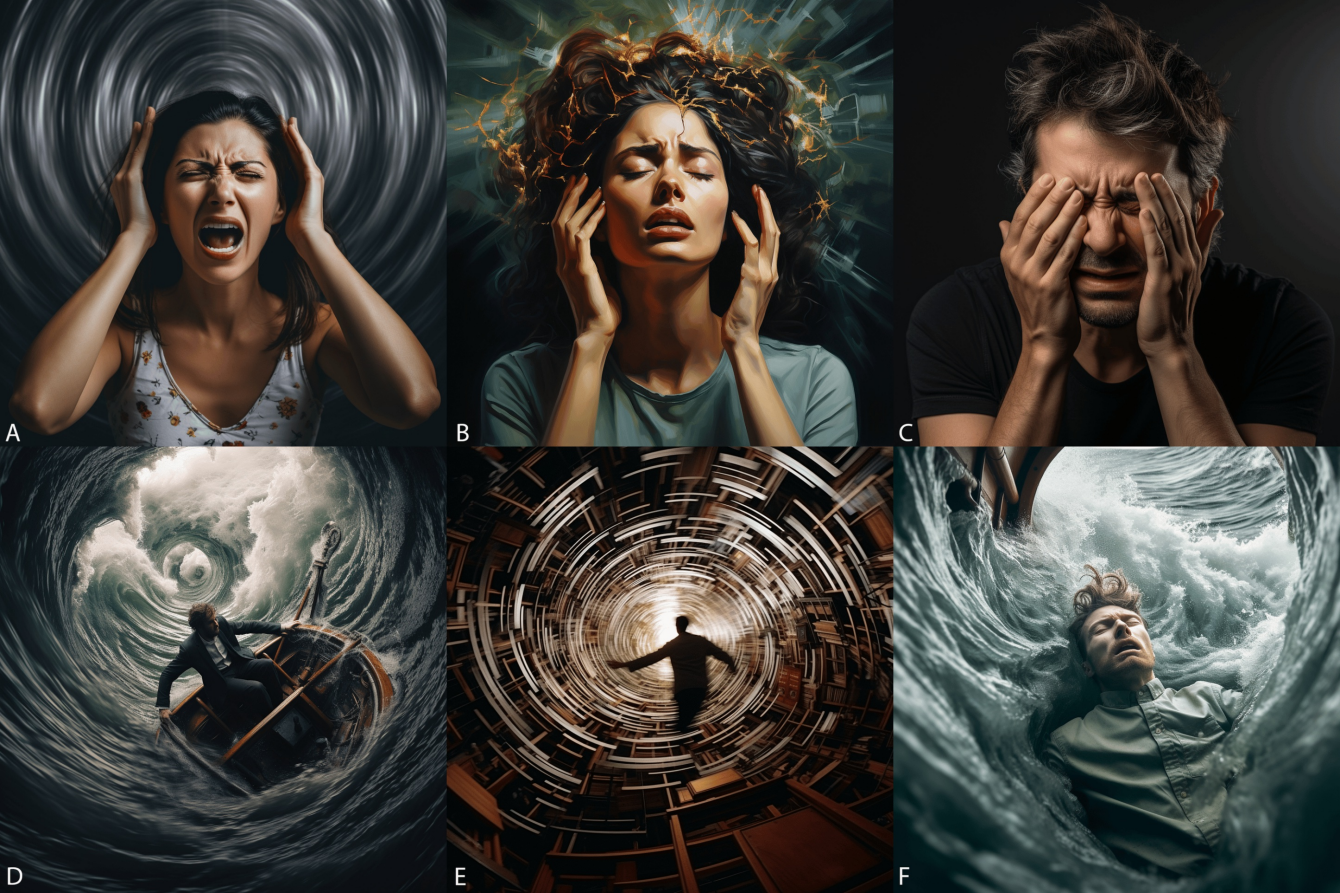
Head and neck symptoms (images generated by Midjourney) A) tinnitus, B) migraine headache, C) sinus pressure, D) mal de debarquement, E) vertigo, and F) sea sickness.

**Figure 5:**
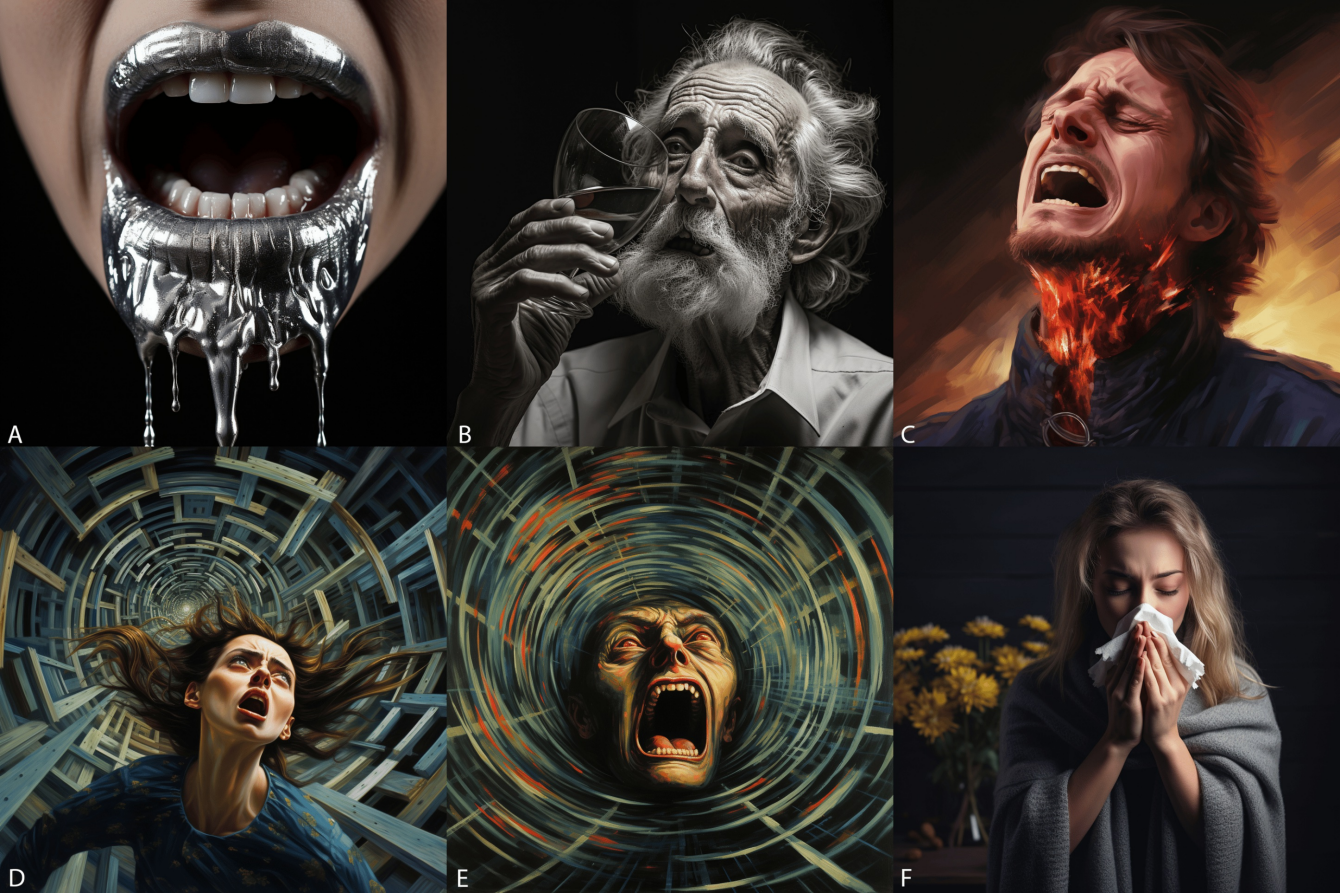
Head and neck symptoms (images generated by Midjourney) A) Dysgeusia (metallic taste), B) xerostomia (dry mouth), C) odynophagia (sore throat), D) dizziness, E) bloodshot eyes with thunderclap headache (worst headache of life), and F) seasonal allergies.

**Figure 6:**
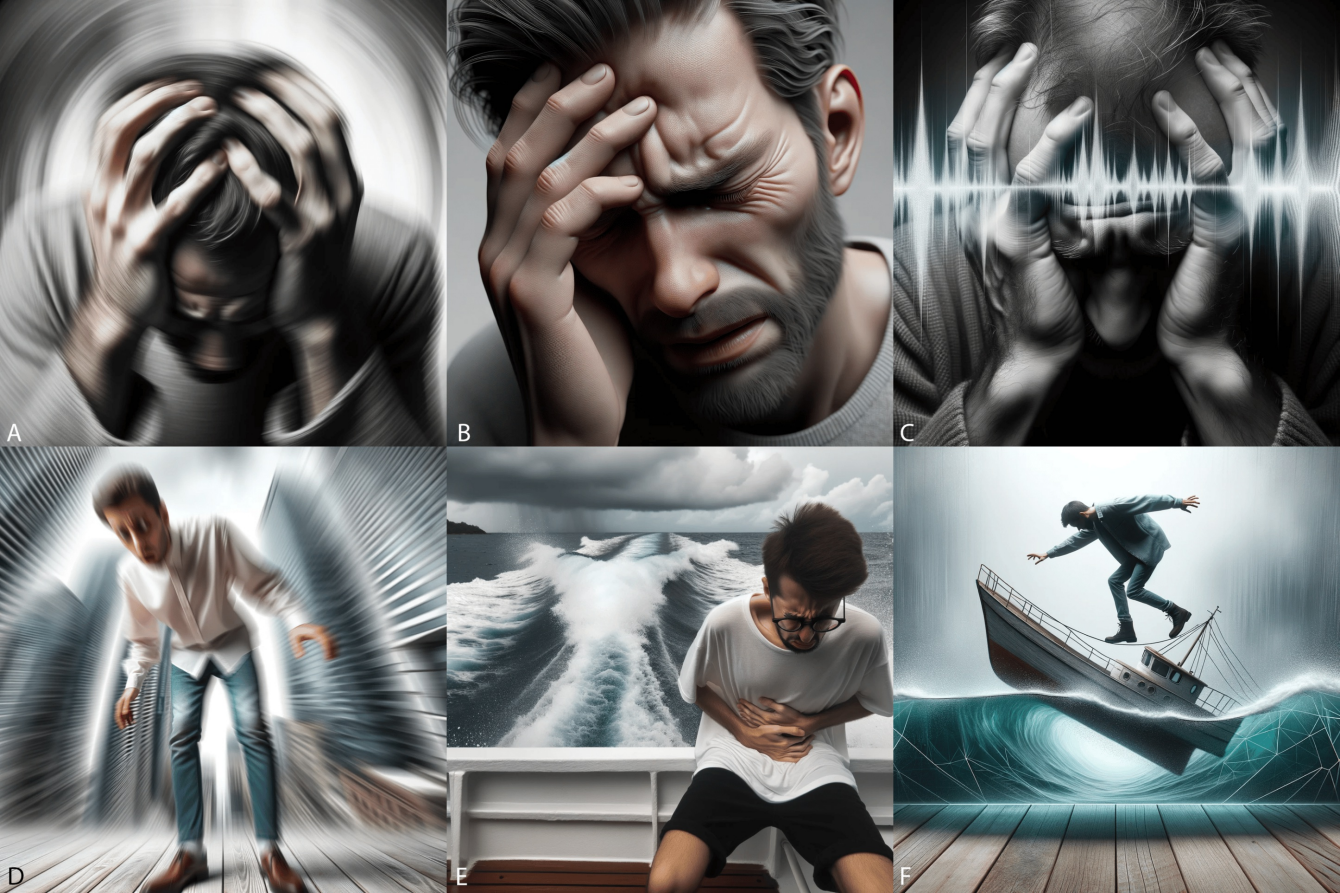
Head and neck symptoms (images generated by DALL-E 3) A) Vertigo, B) headache, C) tinnitus, D) dizziness, E) sea sickness, and F) mal de debarquement (feeling of rocking in a boat).

Initially, the outer, middle, and inner ear images were generated in the style of renowned artists of the past (Figures 1A-1F, 2A-2F, 3A-3F). To illustrate subjective clinical otolaryngology symptoms, the symptom alone was provided as a text prompt to both Midjourney and DALL-E 3 (Figures 4A-4F, 5A-5F, 6A-6F). When needed, more literal, descriptive, and specific terms were added. When text-only prompts were ineffective, such as for the generation of the middle ear, Midjourney was provided with an initial sketch of the ossicles (Figures 2A-2F). A sample very short video clip with sound representing tinnitus (Video 1) was also created for demonstrative purposes from an initial AI-generated image in Midjourney within a web-based video generator called Kaiber.ai. The audio was acquired separately from an online repository of sounds, Pixabay.

**Video 1:** Tinnitus A video created using Kaiber.ai with an AI-generated image from Midjourney v5.2 using the prompt "a man screaming in discomfort because of ringing sound in ears, hands on ears, mouth wide open silently screaming, forehead frown lines, in the style of realism" combined with audio from Pixabay.

Potential applications in clinical settings

Improved Patient Communication

AI-generated images using such descriptions may help patients who struggle to describe accurate narratives of their experiences including those with cognitive impairments, language barriers, cognitive deficits, cultural differences, or overall, difficulty expressing their subjective experiences and perceptions. Toddlers and children may also have difficulty describing their symptoms. When not addressed effectively, communication barriers may contribute to disproportionately negative clinical outcomes and healthcare inequities affecting minority populations [4].

Clinical Diagnosis

Text-to-image programs may be particularly useful for transforming poorly understood subjective symptoms that are characterized by varying descriptions into vibrant illustrations that can be physically or digitally presented to the patient. Such symptoms, for instance, in the otologic setting may include tinnitus and vertigo.

Algorithmic AI Diagnostic Assistant Tool

Ultimately, a patient may choose a multitude of images combined with text that may be representative of their symptoms and an AI algorithm can suggest a set of differential diagnoses to the clinical team [5]. In conjunction with generative AI models such as LLMS, this can help detect unique diseases and more effectively guide clinical outcomes.

Enhanced Patient Education and Compliance

AI image generators can revolutionize patient-clinician interactions by producing tailored visual aids that resonate with individual patients. By visualizing complex medical concepts or conditions, these tools can bridge communication gaps, ensuring patients have a clear understanding of their health status and the proposed interventions, fostering trust and collaboration in the care process and potentially improving compliance and adherence to medical advice [3].

Patient Expression, Support, and Coping

Beyond the clinical domain, AI image generators can be therapeutic tools for patients. By allowing patients to visually express their feelings, fears, or experiences, these tools may offer patients emotional comfort and catharsis. Visual representations can also be used in support groups, helping patients relate to and cope with shared experiences, fostering a sense of community and understanding [2].

Future directions and challenges

As these tools improve in their ability to create more accurate human anatomy, they become more valuable in illustrating pathophysiology, surgical and procedural techniques, disease progression and treatment response. Even more impactful will be AI-generated videos for interactive medical education of trainees and patients.

When using AI-generated images in clinical settings, implicit biases relating to gender, race, body type, and other factors must be considered thoughtfully. While images may enrich dialogues, they cannot replace clinical judgment. Copyright and source acknowledgement remain evolving areas, with services like DALL-E 3 allowing opt-outs. Training the models on medically vetted images could drastically improve accuracy and realism. As video generation advances, ethical concerns around deepfakes and misinformation intensify. Awareness of generative AI's remarkable capabilities and limitations is vital for responsible usage and a collaborative effort among regulators, ethicists, artists, clinicians, and technologists is needed to ensure responsible, equitable usage as these technologies progress rapidly. The potential is immense, but education and oversight are imperative.
